# Takotsubo cardiomyopathy after cardiac surgery: A case-series and systematic review of literature

**DOI:** 10.3389/fcvm.2022.1067444

**Published:** 2023-01-10

**Authors:** Driss Laghlam, Olivier Touboul, Morgane Herry, Philippe Estagnasié, Jean-Claude Dib, Mohamed Baccouche, Alain Brusset, Lee S. Nguyen, Pierre Squara

**Affiliations:** Department of Cardiology and Critical Care, Clinique Ambroise Paré, Neuilly-sur-Seine, France

**Keywords:** takotsubo cardiomyopathy, cardiac surgery, mitral valve surgery, tricuspid valve surgery, cardiogenic shock

## Abstract

**Background:**

Takotsubo cardiomyopathy (TTC) is a rare entity after cardiac surgery.

**Aims:**

To describe patients’ profile who developed postoperative TTC after cardiac surgery, management, and outcomes.

**Methods:**

We performed a systematic literature search to extract cases of TTC after adult cardiac surgery (from 1990 to 2021). Additionally, we extracted all cases of TTC in a prospective single-center cohort database of 10,000+ patients (from 2007 to 2019). We then combined all cases in a single cohort to describe its clinical features.

**Results:**

From 694 screened articles, we retained 71 individual cases published in 20 distinct articles (19 cases reports and 1 case-series). We combined these to 10 cases extracted from our cohort [among 10,682 patients (0.09%)]. Overall, we included 81 cases. Patients were aged 68 ± 10 years-old and 64/81 (79%) were women. Surgery procedures included mitral valve and/or tricuspid valve surgery in 70/81, 86%. TTC was diagnosed in the first days after surgery [median 4 (1–4) days]. Incidence of cardiogenic shock, defined as requirement of vasopressor and/or inotropic support was 24/29, 83% (data available on 29/81 patients). Refractory cardiogenic appeared in 5/81, 6% who required implantation of arterio-venous extra-corporeal membrane oxygenation, and 6/81, 7%, intra-aortic balloon pump. In-hospital mortality was 5/81, 6%.

**Conclusion:**

This systematic review, based on case reports and case series, showed that postoperative TTC appears as a rare complication after cardiac surgery and mainly occurred after mitral and/or tricuspid valve repair procedures. In this population, TTC is associated with high rate of cardiogenic shock.

## Graphical Abstract

Takotsubo cardiomyopathy after cardiac surgery: Combined case-series and systematic review of literature. 
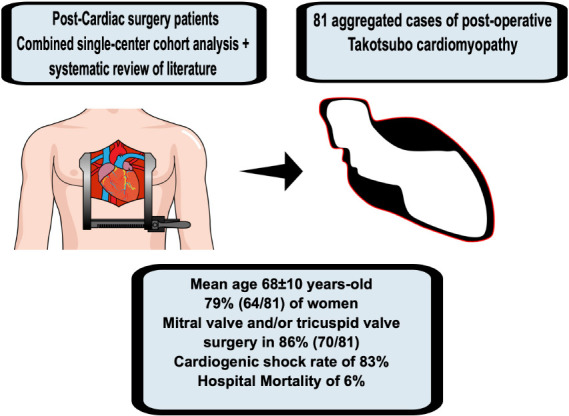

## Highlights

- Takotsubo cardiomyopathy (TTC) is a rare complication after cardiac surgery.
- Takotsubo cardiomyopathy involved primary mitro-tricuspid procedure.
- High rate of TTC-related cardiogenic shock in cardiac surgery settings.

## Introduction

Takotsubo cardiomyopathy (TTC) is a transient myocardial dysfunction syndrome, so named because the appearance of the left ventricle look like the traditional Japanese octopus jar with a narrow neck and round bottom, due to its pathognomonic echocardiographic features of apical ballooning (1). TTC are defined by strict criteria which include a trigger (physical or emotional stress), specific echocardiographic findings, ECG features and the exclusion of differential diagnoses (2). Among hypothesized mechanics, the role of endogenous catecholamines, microvascular disorders, hormonal and genetic factors have been suggested. Risk factors include female sex, age, and neurological or psychological conditions (2). The estimated incidence is 2% of all troponin-positive patients presenting with suspected acute coronary syndrome, with a plausible underestimation of the phenomenon (3). Although ventricular dysfunction due to TTC is usually reversible within a few days to weeks, in-hospital mortality is 4.5%, related to cardiogenic shock and extracardiac consequences (4).

Postoperative TTC after cardiac surgery have seldomly been reported. In the present work, we aim to investigate the incidence of TTC after cardiac surgery, its risk factors, and subsequent outcomes.

## Methods

In a single-centre cohort study, we retrospectively screened patients who underwent cardiac surgery with cardiopulmonary bypass from January 2007 to March 2019 using a prospectively collected database, including 35 perioperative data. The cohort study was approved by our local institutional review board committee. Data are part of the REGISTRY for the improvement of Postoperative OutcomeS in Cardiac and Thoracic Surgery (RIPOSTE) database (NCT03209674). Since this study was observational, the need for written consent was waived according to French Law (Law 88-1138 relative to Biomedical Research of December 20, 1988, modified on August 9, 2004). Lack of patients’ opposition to the use of data was systematically sought for.

Patients who had previously had TTC were not involved in setting the research question or the outcome measures.

Takotsubo cardiomyopathy diagnosis was defined as the association of an acute left ventricular dysfunction with focal wall abnormalities extended beyond a single coronary artery that recovers spontaneously within days or weeks, repolarization abnormalities on ECG (ST-segment elevation or depression, T-wave inversion and/or prolongation in the QTc interval), modest increases in CPK and troponin and the exclusion of differential diagnoses such as a culprit coronary obstruction, a myocarditis, or a pheochromocytoma (2).

In patients in whom TTC diagnosis was retained, ECG, echocardiography, and preoperative coronary angiography were systematically reviewed and described.

### Literature review

A literature search was started on June 13, 2021, and completed on August 13, 2021, using the National Library of Medicine. The systematic review was not registered. We created and followed the Preferred Reporting Items for Systematic Reviews and Meta- Analysis checklist protocol (5). The search was developed for the National Library of Medicine using a combination of subject headings and free-text terms: *[(takotsubo OR stress cardiomyopathy OR broken heart) AND (surgery OR post-operative)] AND (cardiac OR mitral valve OR aortic valve OR tricuspid valve OR coronary bypass grafting OR ascending aorta).* All dates of publication prior to 1990 [date of first described TTC (1)] were excluded. Articles were excluded if not published in English. Only research concerning adults’ subjects were included. We included all available literature that reported occurrence and outcomes of TTC after any cardiac surgery procedure.

The search (National Library of Medicine) provided initially resulted in the 694 studies. Articles and abstracts were considered eligible for inclusion if they: (1) described very specific clinical scenario of TTC after a cardiac surgery and (2) reported whether patient’s characteristics and outcomes. Thirty-seven full-text articles were assessed for eligibility. Finally, we included 20 studies of 71 cases in this review.

## Results

### Registry case extraction

**FIGURE 1:**
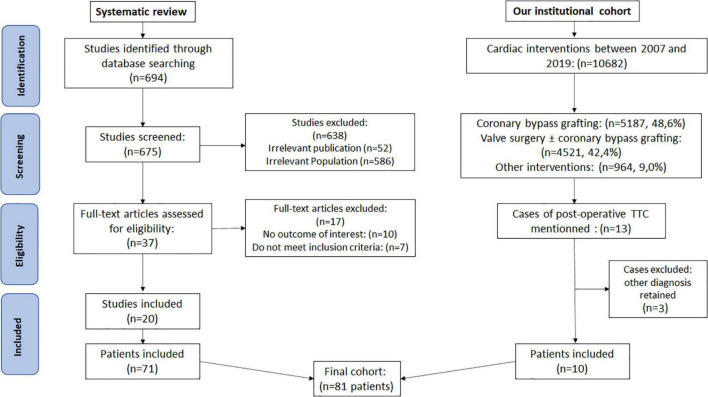
PRISMA flowchart for the systematic review and flow chart of our institutional cohort.

Between 1st January 2007 and 1st March 2019, 10,682 cardiac surgery procedures were performed, including 5,187 coronary artery bypass graft procedures; 4,531 valve procedures (with or without cardiopulmonary bypass), including 1,114 mitral valves; and 964 other interventions. We finally retained 10 cases of TTC (see flow chart, Figure 1). Baseline preoperative characteristics and outcomes are presented in Table 1.

**TABLE 1:** Characteristics and outcomes of our institution’s post-operative takotsubo cohort.

Patient	Age	Gender	BMI	Euroscore 2 (%)	Pre-op angio	Type of surgery	CPB (min)	ACC (min)	LVEF D-1/D0/D7 (%)	ECG T-wave < 0	Vasoactives amines duration (days)	Mechanical assistance	ICU duration (days)	Hospitalization duration (days)	Death
1	73	F	22	8.6	N	MVR + TVP	65	55	65/20/52	Global	13	–	21	49	Yes
2	61	F	20	4.7	N	TVP (redux)	44	30	65/25/60	Anterolateral	4	–	6	15	No
3	81	F	20	5.9	N	MVP	67	60	75/50/73	Global	4	–	7	12	No
4	61	F	20	1.1	N	MVP	305	180	65/20/60	Anterolateral	2	–	8	10	No
5	71	M	20	3.6	N	MVP	111	62	65/30/70	Apicolateral and inferior	11	IABP	18	31	No
6	64	F	18	3.5	N	MVR	74	58	67/25/12	Global	6	ECMO	6	6	Yes
7	80	F	28	4	N	MVR	73	55	70/20/41	Global	3	–	3	10	No
8	86	F	22	7.5	N	MVP	88	66	64/30/-	Anterior	3	–	3	3	Yes
9	78	F	18	6.1	N	MVR	67	48	77/36/65	Inferolateral	5	–	5	17	No
10	71	F	20	5.3	N	MVR	76	51	55/27/50	Global	8	–	9	17	No

ACC, aortic cross clamping time; BMI, body mass index; CPB, cardiopulmonary bypass time; D-1, day before surgery; D0, day of surgery; D7, day seven after surgery; ECG T-waves < 0, description of territory of new T-waves negativity on electrocardiogram; ECMO, extracorporeal membrane oxygenation; IABP, intra-aortic balloon pump; ICU, intensive care unit; LVEF, left ventricular ejection fraction; MVR, mitral valve replacement; MVP, mitral valvuloplasty; N, normal; Pre-op angio, pre-operative coronary angiography; TVP, tricuspid valvuloplasty.

### Literature case extraction

From 694 articles, we retained 71 individual cases published in 20 distinct articles, between 2007 and 2021 (6–25) (see flow chart, Figure 1; literature cases are presented in Supplementary Table 1 with ratings quality of the evidence of 1–5 from the Oxford Centre for Evidence-based Medicine for ratings of individual studies).

### Aggregated cases analysis

#### Clinical presentation

Baseline characteristics, reason for surgery, type of surgery, and outcomes of the aggregated cohort are displayed in Table 2.

**TABLE 2:** Characteristics and outcomes of whole post-operative takotsubo cohort.

	Overall cohort (*n* = 81)
**Baseline characteristics**	
Age, mean ± SD	68 ± 10.0
Women, *n* (%)	64 (79)
Hypertension, *n* (%)	12 (15)
Diabetes, *n* (%)	7 (8.6)
Previous ischemic cardiopathy, *n* (%)	2 (2.4)
Pre-operative LVEF(%), median (IQR), 80/81	60 (60–65)
**Indication for surgery** **Available on 81/81 patients**	
Isolated severe mitral regurgitation, *n* (%)	31 (38)
Isolated severe mitral stenosis, *n* (%)	12 (15)
Mixed severe mitral disease, *n* (%)	11 (14)
Isolated severe aortic stenosis or regurgitation, *n* (%)	6 (7)
Isolated severe tricuspid regurgitation	5 (6)
Combined severe mitral regurgitation + aortic valvulopathy, *n* (%)	6 (7)
Combined severe mitral + tricuspid regurgitation, *n* (%)	2 (2)
Combined severe mixed mitral disease + tricuspid regurgitation, *n* (%)	2 (2)
Combined severe mitral stenosis + coronary bypass grafting, *n* (%)	1 (1)
Aortic regurgitation + coronary bypass grafting, *n* (%)	1 (1)
Aorta, *n* (%)	1 (1)
Other, *n* (%)	3 (4)
**Surgery** **Available on 81/81 patients**	
Isolated mitral valve surgery, *n* (%)	54 (67)
Isolated mitral valve replacement, *n* (%) (29/81)	12 (41)
Isolated mitral valve repair, *n* (%) (29/81)	6 (21)
Isolated tricuspid valve surgery, *n* (%)	5 (6)
Isolated aortic valve surgery, *n* (%)	6 (7)
Combined mitral plus aortic valve surgery, *n* (%)	6 (7)
Combined mitral plus tricuspid valve surgery, *n* (%)	4 (4)
Combined coronary bypass grafting + aortic valve surgery, *n* (%)	1 (1)
Combined coronary bypass grafting + mitral valve surgery, *n* (%)	1 (1)
Aorta surgery, *n* (%)	1 (1)
Other, *n* (%)	3 (4)
Cardiopulmonary bypass time, median (IQR), 74/81	90 (73–128)
Aortic cross clamping time, median (IQR), 74/81	65 (55–80)
**Outcomes**	
Post-operative day of diagnosis, median (IQR), 80/81	4 (1–4)
LVEF on diagnosis (%), median (IQR), 27/81	26.5 (20–30)
LVEF on discharge (%), median (IQR), 27/81	60 (50–60)
**Vasoactive drugs, 81/81**	
Norepinephrine, *n* (%)	34 (42)
Epinephrine, *n* (%)	28 (35)
Dopamine, *n* (%)	26 (33)
Dobutamine, *n* (%)	38 (47)
Arterio-venous extracorporeal membrane oxygenation, *n* (%)	5 (6)
Intra-aortic balloon pump, *n* (%)	6 (7)
Mortality, *n* (%)	5 (6.3)
Intensive care unit length of stay, median (IQR), 69/81	7.5 (5–12)

Among 81 cases of definitive postoperative TTC, 64/81, 79% were women, and the mean age was 68 ± 10.0 years. They showed normal preoperative coronarography and median preoperative left ventricular ejection fraction was preserved 60 (60–65)% (data available in 80/81, 99%).

The reason for surgery (data available in 81/81, 100%) was the treatment of atrioventricular ventricular severe valvulopathy in 70/81, 86% cases; including isolated severe mitral regurgitation in 31/81, 38% cases, isolated severe mitral stenosis in 12/81, 15% cases, mixed severe mitral disease (severe mitral stenosis associated with severe insufficiency) in 11/81, 14% patients, isolated severe tricuspid regurgitation in 5/81, 6% patient, combined severe mitral regurgitation associated with aortic valvulopathy in 6/81, 7% patients, and combined severe mitral stenosis or regurgitation associated with severe tricuspid regurgitation in 4/81, 5% cases.

The characteristics of the surgical procedures are presented in Table 2. All procedures were elective (no emergencies). No patient received catecholamines before surgery. The median cardiopulmonary bypass and aortic cross clamping durations were (data available in 74/81, 91%): 90 (73–128) minutes and 65 (55–80) minutes, respectively. Most procedures (data available in 81/81, 100%) involved mitral valve or tricuspid valve surgery in 70/81, 86% cases; including isolated mitral valve surgery in 54/81, 67%, isolated tricuspid valve surgery in 5/81, 6%, combined mitral plus tricuspid or aortic valve surgery in 10/81, 12%, and one combined mitral valve surgery plus coronary bypass grafting, 1%.

#### Assessment and diagnosis

Coronary angiography was performed in 7/29, 24% patients and did not reveal culprit lesions (information on coronary angiography, whether it was performed or why it would not, was available in 29/81, 36%). In other patients, based on the normal preoperative coronarography, and the typical wall motion abnormalities, the diagnosis of acute myocardial infarction was judged to be unlikely by authors; consequently, coronary angiography was not performed.

Postoperative outcomes are presented in Table 2. Diagnosis of TTC was made on day 4 (1–4) after surgery. Whenever described (27 available data), concomitant left ventricular dysfunction was always observed with a median LVEF of 26.5% (20–30).

#### Treatment

Incidence of cardiogenic shock was 24/29, 83% (information on incidence of cardiogenic shock was available in 29/81, 36%). Management of TTC (data available in 81/81, 100%), required use of vasopressor and/or inotropic support due to cardiogenic shock by norepinephrine in 34/81, 42%, epinephrine in 28/81, 35%, dopamine in 26/81, 33% and dobutamine in 38/81, 47% of patients. Refractory cardiogenic shock requiring implantation of arterio-venous extra-corporeal membrane oxygenation appears in 5/81, 6%, patients, and 6/81, 7%, needs intra-aortic balloon pump.

#### Prognosis

Subsequent in-hospital mortality was 5/81, 6%. Intensive care length of stay was 7.5 (5–12) days after surgery (data available in 69/81, 85%). There was recovery in most cases before the end of the hospitalization period, as LVEF was 60 (50–60)% on discharge (data available in 27/81, 33%).

## Discussion

In this study which combined cases extracted from a large registry and a systematic literature search, we found eighty-one cases of TTC following cardiac surgery. Although the incidence seems to be low, 83% of patients developed cardiogenic shock requiring vasopressors and/or ionotropic agents; and mortality was 5/81, 6%. Of note, most procedures involved mitral or tricuspid valve repair or replacement.

Because TTC is usually associated with transient LVEF decrease, with quick recovery, the prognosis has long been supposed to be benign. However, more recent studies demonstrated that subsequent in-hospital mortality reaches 4.5%, which is equivalent to the STEMI one (4). In this context, we found a subsequent mortality was 6% in TTC after cardiac surgery. Postoperative TTC had a low incidence (0.1% in our institutional cohort) and affected women in 79% of cases in whole cohort, which is consistent with the at-risk population described so far. TTC occurs early after surgery; but we found two case of late-onset TTC (11, 16).

The association between TTC and atrioventricular valvular procedures (80% mitral, 11% tricuspid) suggests a possible involvement of the type of cardiac procedure which was performed. First, a plausible mechanism may be the abrupt change in ventricular loading conditions when chronic atrioventricular regurgitation is suddenly corrected, may lead to a parallel sharp increase in endogenous catecholamines, independent of the surgical conditions. As described, TTC is initiated by an increase in plasmatic catecholamine levels, with evidence of cardiac sympathetic hyperactivity on iodine-123–meta-iodobenzylguanidine (I-MIBG) scintigraphy (26). Therefore, TTC may also be related to an adrenergic response to a sudden ventricular load mismatch. Consequently, reasoned use of catecholamines in TTC has therefore been suggested. Myocardial fibers are not uniformly receptive to hormones (27), and catecholamines, due to a regional distribution of sympathetic adrenoreceptors (28). This asymmetry is thought to explain the region wall motion abnormalities encountered in TTC, the most prominent being apical ballooning. Second, chronic atrioventricular regurgitations, be they mitral or tricuspid, cause progressive ventricular and atrial enlargement through altered protein expression, sarcomere remodeling and myocyte elongation leading to self-sustaining dilatation and regurgitation worsening (29–31). When indicated, valve surgery aims to restore valve competency, and abruptly terminating retrograde flow due to regurgitation. In the specific case of chronic severe atrioventricular repairs, the sudden decrease in ventricular preload and increase in afterload, leads to a mismatch in myocardial mechanics, peak in ventricular myocardial stress and transient increase in myocardial O_2_ consumption. These elements may be very specific to mitral and tricuspid procedures, as all other cardiac surgery procedures usually put ventricles in a more favorable physiological environment as compared to the preoperative state, as demonstrated by *in silico* modelization. Briefly, sudden correction of mitral regurgitation increased left and right ventricular fiber stress by 40 and 15%, respectively, whereas tricuspid regurgitation correction increased left and right ventricular fiber stress by 26 and 19%, respectively (32). Concordant data were found in cardiac magnetic resonance imaging experimentation showing significant decreases in end-diastolic, end-systolic, and stroke volumes after mitral valve surgery, which were associated with a significant postoperative decrease of mean kinetic energy and systolic and early diastolic kinetic energy peaks (33).

However, we showed that post cardiac surgery TTC did not occurred only after mitral or tricuspid procedure; therefore, the physiopathology is not unequivocal.

Finally, the involvement of inflammation, which may be related to cardiopulmonary bypass and usual cardiac postoperative state, may be an additional risk, albeit, impossible to analyze independently. Indeed, inflammation has previously been related to TTC, with the participation of systemic inflammatory cells (with a higher proportion of classical CD14^++^CD16^–^ and lower proportion of non-classical CD14^++^CD16^+^ expressing monocytes in patients with TTC) and cytokines (higher levels of interleukin-6 and CXCL1 chemokines in patients with TTC) (34). While we did not assay our patients, thus, cannot confirm these same assumptions; CPB-related inflammation has also been associated with monocyte activation (35) and interleukin-6 elevation (36). To that extent, CPB-related inflammation may be considered akin to a minor form of cytokine release syndrome, which has extensively been associated with TTC in other settings than cardiac surgery (37–39).

We reported here a high rate of cardiogenic shock complicating TTC after cardiac surgery. As of now, acute heart failure is the most common complication of TTC in non-surgery patients (29%) (40) but cardiogenic shock account for only 5–6.1% (40, 41), and mortality from 2.6 to 14.5% (40–42). Meanwhile, after in surgery patients, 0.2–10% of those undergoing coronary or valvular cardiac procedures develop post cardiotomy cardiogenic shock (43, 44). This discrepancy between cardiogenic shock rate in TTC following cardiac surgery and in those in medical settings, could be explained by factors who potentialized cardiac dysfunction such cardiotomy and cardiopulmonary bypass induced inflammation. Furthermore, the incidence of cardiogenic shock could be overestimated by the retrospective character of the studies included with a possible selection bias with only the most serious cases reported in the literature; and the unavailability of the incidence of cardiogenic shock in the case series of 52 patients.

A strength of our study lies on the stringent criteria used to define TTC. TTC after cardiac surgery must be distinguished from other postoperative complications. Indeed, in the context of cardiac surgery, confounding factors may be incriminated toward misdiagnosis TTC. Lack of myocardial protection, coronary embolism, coronary vasospasm (45) and exogenous catecholamine-mediated toxicity are indeed plausible (46) (of note, in this cohort, no patient received catecholamines before surgery). Abnormalities on ECG or/and on echocardiography A in a coronary territory must direct toward a coronary origin or a gas embolism of the left ventricular dysfunction, whereas a diffuse left ventricular dysfunction could direct rather toward a lack of myocardial protection during cardiopulmonary bypass.

The troponin elevation is unspecific in cardiac surgery, since the procedure itself induces an elevation, even in the absence of postoperative cardiac complications.

Specifically, regarding mitral valve surgery, the loss of ventricular-annular continuity observed when both leaflets and papillary muscles are excised is another possible cause of left ventricular dysfunction. However, the preservation of the posterior mitral leaflet and its subvalvular apparatus is recommended when possible (47). In our institutional patients, 3 out of 10 had partial resection of the posterior mitral leaflet, which may have contributed to the left ventricular dysfunction.

We acknowledge several limitations. Our cohort was a single-center retrospective case-series. Given the conflicting factors in this context, the diagnosis of TTC could be difficult and although the data collection was prospective, the TTC incidence could be underestimate. Regarding the systematic review of the literature, we found only case reports and one case-control study with limited data on some valuable data; increasing the risks of confounding and bias. In the lack of large study and based only on case reports, this study could reflect imperfectly the real prevalence and course of post cardiac surgery TTC patients.

## Conclusion

In this study which combined cases extracted from a large registry and a systematic literature search, we confirmed the existence of TTC following cardiac surgery and observed that TTC occurred preferentially after mitral and tricuspid procedures.

## Data availability statement

The raw data supporting the conclusions of this article will be made available by the authors, without undue reservation.

## Ethics statement

The studies involving human participants were reviewed and approved by the Clinique Ambroise Paré Ethics Committee. The patients/participants of our cohort provided their written informed consent for the use of their de-identified data.

## Author contributions

DL, OT, and LN have substantially contributed to the conception and the design of the study and were major contributors in writing the original manuscript. LN, PE, and PS have also substantially contributed to the conception and the design of the study and were contributors in revision of the manuscript. DL, MB, OT, MH, AB, and J-CD have substantially contributed to the acquisition and analysis of the data. All authors read and approved the final manuscript.
