# Supplementary material for: Takotsubo cardiomyopathy after cardiac surgery: A case-series and systematic review of literature
Source: Front Cardiovasc Med. 2023 Jan 10;9:1067444. doi: 10.3389/fcvm.2022.1067444 (PMC9871635; doi:10.3389/fcvm.2022.1067444)
Supplement: Supplementary file 1 [file Table_1.DOCX]

Postoperative Takotsubo cardiomyopathy after cardiac surgery: a case-series and systematic review of literature

Authors: Driss Laghlam*, MD; Olivier Touboul*, MD; Morgane Herry, MD; Philippe Estagnasié, MD; Jean-Claude Dib, MD; Mohammed Baccouche, MD; Alain Brusset, MD; Pierre Squara, MD and Lee S. Nguyen, MD, PhD

*equally contributed to this manuscript

From the Department of Cardiology and Critical Care, Clinique Ambroise Paré, 27 boulevard Victor Hugo, 92200, Neuilly-sur-Seine, France

Corresponding author:

Dr Driss Laghlam

Research & Innovation Department, RICAP

CMC Ambroise Paré

27 boulevard Victor Hugo, 92200, Neuilly-sur-Seine, France

Email: driss.laghlam@gmail.com

Phone +33664435655

**Supplemental Table 1: Extracted cases from literature of Takotsubo cardiomyopathy following cardiac surgery**

| Author (year)  Country | Age (years-old)/Gender | Type of Surgery | Intra-operative times  (min) | Clinical presentation | Onset  LVEF | Vasoactives amine support | Mechanical assistance | Outcome  LEVF on discharge | Quality  of the evidence |
| --- | --- | --- | --- | --- | --- | --- | --- | --- | --- |
| Itoh H. (2007)  Japan ^1^ | 59/ Women | MVP | CPB: 242  ACC: 62 | CS at Day 0 | NR | Dobutamine + Dopamine | IABP | Favourable  NR | 5 |
| Kogan A. (2008)  Israel ^2^ | 62/ Women | MVR+ TVP | CPB:153  ACC: 101 | CS at Day 0 | 35% | Dobutamine + Epinephrine + Norepinephrine | None | Favourable  55% | 5 |
| Blazquez J. (2010) Spain ^3^ | 68/ Women | MVR | CPB: 83  ACC: 68 | CS at Day0 | 20% | Dobutamine | IABP | Favourable  65% | 5 |
| Yamane K. (2011) USA ^4^ | 68/ Women | MVR+ TVP | CPB: 102  ACC: 73 | CS at Day 0 | 30% | Dobutamine +  Epinephrine | IABP | Favourable  Normal | 5 |
| Gariboldi V. (2011) France ^5^ | 66/ Women | MVR | CPB: 103  ACC: 80 | CS at Day 0 | 20% | Dobutamine +  Norepinephrine | None | Favourable  Normal | 5 |
| Farber G. (2011)  Germany ^6^ | 69/ Women | MVR | CPB:133  ACC: 78 | CS at Day 14 | 15% | Epinephrine | IABP | Favourable  60% | 5 |
| Vernick W. (2012) USA ^7^ | 78/Men | MVR+ TVP | NR | CS at Day 0 | 20% | Epinephrine +  Norepinephrine | None | Favourable  60% | 5 |
| Attisani (2013)  Italy ^8^ | 77/ Women | MVR+Maze | NR | CS at Day1 | 20 | NR | None | Favourable  60% | 5 |
| Wang (2013)  Australia ^9^ | 77/ Women | AVR+ CABG | NR | CHF |  | None | None | Favourable  60% | 5 |
| Li S. (2014)  USA ^10^ | 37/ Women | MVR | CPB: 92  ACC: 82 | CS at Day 0 | 20% | Epinephrine +  Norepinephrine | ECMO | Favourable  60% | 5 |
| Lorca R. (2015)  Spain ^11^ | 68/ Women | MVR+ AVR | NR | CHF at Day 30 | 35% | None | None | Favourable  Normal | 5 |
| Pergolini A. (2015) Italy ^12^ | 66/ Women | MVR | CPB :133  ACC :82 | CHF at Day4 | 30% | None | None | Favourable | 5 |
| Chiariello G.A (2016) Italy ^13^ | 72/ Women | Aortic/RA fistula repair+ TVP | NR | CHF | 29% | None | None | Favourable  46% | 5 |
| Garcia-Delgado (2016) Spain ^14^ | 68/ Women | Left atrium Myxoma Resection | CPB:83  ACC:52 | Cardiac arrest at Day1 | 20% | Dobutamine + Epinephrine + Norepinephrine | ECMO | Favourable  50% | 5 |
| Elikowski W. (2019) Poland ^15^ | 78/ Women | MVR+ CABG | CPB : 103  ACC: 73 | CS at Day 1 | 28% | Nad | None | Favourable  60% | 5 |
| Choi J. (2020) ^16^  Korea | 52/Women | MVR+Maze | NR | CS at Day 2 | 26% | Dobutamine + Epinephrine + Norepinephrine | ECMO | Favourable  58% | 5 |
| Devesa A. (2020) Spain ^17^ | 79/Women | MVR | NR | CS at Day 1 | 20% | Dobutamine + Nad | IABP | Favourable  50% | 5 |
| Vahzev Z. (2020) Bulgaria ^18^ | 57/Men | MVR+AVR | CPB :96  ACC :63 | CS at Day1 | 28% | Dobutamine + Dopamine | None | Favourable  48% | 5 |
| Totaro P. (2020)  Italy ^19^ | 67/Women | MVR | NR | CHF at Day 3 | 30% | None | None | Favourable  60% | 5 |
| Kim Y.S. (2021) ^20^  Cohort of 52 TTC over 10years  Korea | 55.8±14.4  39/52 (75%) Women | 6/52 isolated AVR  36/52 isolated MVR  4 combined AVR+MVR  4 tricuspid surgery  2 others | CPB 172.3 ± 66.7min  ACC 106.5 ± 49.1min |  |  | Epinephrine: 15/52  Norepinephrine: 24/52  Dobutamine: 23/52  Dopamine: 24/52 | 1/52 | 2/52 early death  Mean ICU stay 8.0 ± 15.2 days | 4 |

1. Itoh H, Miyake Y, Hioki I, Tanaka S, Okabe M. Report of takotsubo cardiomyopathy occurring during cardiopulmonary bypass. *J Extra Corpor Technol.* 2007;39(2):109-111.

2. Kogan A, Ghosh P, Schwammenthal E, Raanani E. Takotsubo syndrome after cardiac surgery. *Ann Thorac Surg.* 2008;85(4):1439-1441.

3. Blázquez JA, González JM, Dalmau MJ, López J. Takotsubo cardiomyopathy after elective mitral valve replacement. *Interact Cardiovasc Thorac Surg.* 2010;11(1):117-119.

4. Yamane K, Hirose H, Reeves GR, Marhefka GD, Silvestry SC. Left ventricular dysfunction mimicking Takotsubo cardiomyopathy following cardiac surgery. *J Heart Valve Dis.* 2011;20(4):471-473.

5. Gariboldi V, Jop B, Grisoli D, Jaussaud N, Kerbaul F, Collart F. Takotsubo syndrome after mitral valve replacement for acute endocarditis. *Ann Thorac Surg.* 2011;91(3):e31-32.

6. Färber G, Mühle A, Doenst T, Borger MA, Mohr FW. Late onset Takotsubo cardiomyopathy after mitral valve replacement. *Thorac Cardiovasc Surg.* 2011;59(8):500-503.

7. Vernick WJ, Hargrove WC, Augoustides JG, Horak J. Takotsubo cardiomyopathy associated with cardiac arrest following cardiac surgery: new variants of an unusual syndrome. *J Card Surg.* 2010;25(6):679-683.

8. Attisani M, Campanella A, Boffini M, Rinaldi M. Takotsubo cardiomyopathy after minimally invasive mitral valve surgery: clinical case and review. *J Heart Valve Dis.* 2013;22(5):675-681.

9. Wang L, Jansz P, Baron D. Delirium and Takotsubo cardiomyopathy following cardiac surgery. *Anaesth Intensive Care.* 2013;41(3):437-438.

10. Li S, Koerner MM, El-Banayosy A, Soleimani B, Pae WE, Leuenberger UA. Takotsubo's syndrome after mitral valve repair and rescue with extracorporeal membrane oxygenation. *Ann Thorac Surg.* 2014;97(5):1777-1778.

11. Lorca R, Callejo F, Pun F, et al. Takotsubo syndrome after heart valve surgery. *Int J Cardiol.* 2015;197:254-256.

12. Pergolini A, Zampi G, Casali G, et al. Takotsubo syndrome after mitral valve replacement: case report and brief review of the literature. *J Cardiothorac Vasc Anesth.* 2015;29(2):431-435.

13. Chiariello GA, Bruno P, Colizzi C, Crea F, Massetti M. Takotsubo Cardiomyopathy Following Cardiac Surgery. *J Card Surg.* 2016;31(2):89-95.

14. García-Delgado M, García-Huertas D, Navarrete-Sánchez I, Olivencia-Peña L, Garrido JM. Extracorporeal membrane oxygenation support for Takotsubo syndrome and long QT after cardiac surgery. *Med Intensiva.* 2017;41(7):441-443.

15. Elikowski W, Małek-Elikowska M, Greberski K, et al. Takotsubo syndrome following mitral valve replacement and left anterior descending coronary artery bypass grafting. *Pol Merkur Lekarski.* 2019;46(271):36-41.

16. Choi JH, Oh ID, Shin E, et al. Extracorporeal membrane oxygenation for takotsubo cardiomyopathy that developed after mitral valve replacement. *Acute Crit Care.* 2020;35(1):51-55.

17. Devesa A, Hernández-Estefanía R, Tuñón J, Aceña Á. Takotsubo syndrome after mitral valve surgery: a case report. *Eur Heart J Case Rep.* 2020;4(6):1-5.

18. Vazhev Z, Stoev H. Takotsubo Cardiomyopathy after Elective Aortic and Mitral Valve Replacement. *Folia Med (Plovdiv).* 2020;62(1):204-207.

19. Totaro P, Triolo OF, Argano V. Rare but real: Takotsubo syndrome following mitral valve surgery. *J Card Surg.* 2020;35(3):721-724.

20. Kim YS, Lim JY. Risk factors for Takotsubo syndrome following cardiac surgery: A case-control study. *J Card Surg.* 2021;36(8):2767-2773.
